# The nuts and bolts of PROSPERO: an international prospective register of systematic reviews

**DOI:** 10.1186/2046-4053-1-2

**Published:** 2012-02-09

**Authors:** Alison Booth, Mike Clarke, Gordon Dooley, Davina Ghersi, David Moher, Mark Petticrew, Lesley Stewart

**Affiliations:** 1 Centre for Reviews and Dissemination, University of York, Alcuin B Block, Heslington, York, UK, YO10 5DD; 2 Centre for Public Health, Institute of Clinical Sciences, Block B, Queen's University Belfast, Royal Victoria Hospital, Grosvenor Road, Belfast, UK, BT12 6BA; 3 Metaxis Ltd, Elmbank Offices, Elmbank Court, Main Road, Curbridge, Oxford, UK, OX29 7NT; 4 Research Translation Branch, National Health & Medical Research Council, 16 Marcus Clarke Street, Canberra City ACT 2600, Australia; 5 Department of Epidemiology, Clinical Epidemiology Program, Ottawa Hospital Research Institute, 725 Parkdale Avenue Ottawa, Ontario, Canada, K1Y 4E9; 6 Community Medicine, Faculty of Medicine, University of Ottawa, 451 Smyth Road, Ottawa, Ontario, Canada K1H 8M5; 7 Department of Social and Environmental Health Research, London School of Hygiene and Tropical Medicine, Keppel Street, London, UK WC1E 7HT

**Keywords:** Systematic review protocol, register, PROSPERO

## Abstract

**Background:**

Following publication of the PRISMA statement, the UK Centre for Reviews and Dissemination (CRD) at the University of York in England began to develop an international prospective register of systematic reviews with health-related outcomes. The objectives were to reduce unplanned duplication of reviews and provide transparency in the review process, with the aim of minimizing reporting bias.

**Methods:**

An international advisory group was formed and a consultation undertaken to establish the key items necessary for inclusion in the register and to gather views on various aspects of functionality. This article describes the development of the register, now called PROSPERO, and the process of registration.

**Results:**

PROSPERO offers free registration and free public access to a unique prospective register of systematic reviews across all areas of health from all around the world. The dedicated web-based interface is electronically searchable and available to all prospective registrants. At the moment, inclusion in PROSPERO is restricted to systematic reviews of the effects of interventions and strategies to prevent, diagnose, treat, and monitor health conditions, for which there is a health-related outcome.

Ideally, registration should take place before the researchers have started formal screening against inclusion criteria but reviews are eligible as long as they have not progressed beyond the point of completing data extraction.

The required dataset captures the key attributes of review design as well as the administrative details necessary for registration.

Submitted registration forms are checked against the scope for inclusion in PROSPERO and for clarity of content before being made publicly available on the register, rejected, or returned to the applicant for clarification.

The public records include an audit trail of major changes to planned methods, details of when the review has been completed, and links to resulting publications when provided by the authors.

**Conclusions:**

There has been international support and an enthusiastic response to the principle of prospective registration of protocols for systematic reviews and to the development of PROSPERO.

In October 2011, PROSPERO contained 200 records of systematic reviews being undertaken in 26 countries around the world on a diverse range of interventions.

## Background

Following the 2010 publication of the PRISMA statement advocating registration of systematic review protocols [1,2] and in response to user demand and increased recognition of the importance of accurate prospective registers of research [3], the Centre for Reviews and Dissemination (CRD) at the University of York in England began to develop PROSPERO, an international prospective register of systematic reviews. The objectives were to reduce unplanned duplication of systematic reviews and to provide transparency in the review process with the aim of minimizing reporting bias [4]. The development process recognized both the academic need for a register and the practical requirements of creating and maintaining one, and was able to take advantage of CRD's existing database infrastructure and information technology (IT) platform supporting the Database of Abstracts of Reviews of Effects (DARE), the NHS Economic Evaluations Database (NHS EED) and the Health Technology Assessment (HTA) database [5].

## Methods

A small international advisory group was formed to help guide the development of the register. The members of the group brought systematic review expertise, including Cochrane, Campbell, and the Evidence-based Practice Centre (EPC) program review methods, experience of clinical trials registers, and authorship of the PRISMA statement [1,2].

The advisory group sought the opinions of a wide range of people for whom the register would be relevant, including clinical and academic researchers, commissioners, and journal editors, through an international consultation process. A two-round electronic modified Delphi survey was used to identify the minimum dataset (Required fields) for PROSPERO and to identify what would represent useful but not essential data (Optional fields) [6]. Participants in the survey were also asked their views on aspects of the functionality of the register. The feedback from the Delphi process and pilot testing were used to develop PROSPERO. This article describes the process of registration that is now in place.

## Results and discussion

### Design of the register

**Figure 1:**
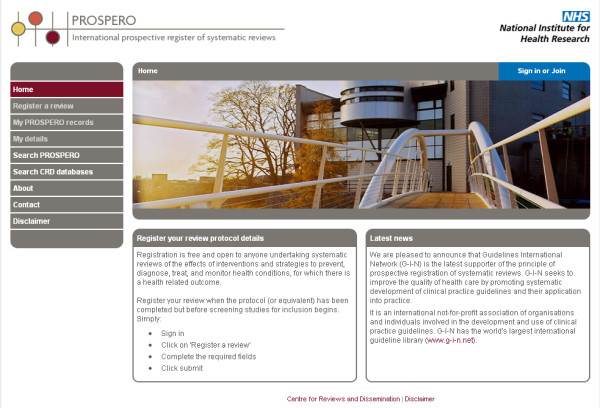
**PROSPERO website homepage**.

The web-based register offers open public access; registering a review and searching the register is free of charge. (Figure 1) The register is electronically searchable; open to all prospective registrants; requires the submission of a minimum data set; and has a validation mechanism to ensure that entries fall within scope and are complete. A unique identification number is issued for each review protocol accepted for registration which becomes part of the review identity and facilitates linkage between the registration record and subsequent publications. PROSPERO records are permanent and an audit trail of any changes to the record is maintained. This allows readers to see how the review has developed or changed over time.

Feedback from many of the 266 participants who completed one or both of the Delphi surveys confirmed the need to balance collection of sufficient information to achieve the objectives of the register, with making sure the registration process was not overly burdensome. The process of registering a review has been made as straightforward, intuitive and user friendly as possible, for example through the use of drop down menus for several items.

A nominated 'Named contact' is responsible for ensuring that the information submitted is accurate and kept up to date, including provision of a link to the report of the review when it is completed. Because detailed information about the planned methods is needed, the Named contact should be the principal investigator or lead researcher, but is not necessarily the 'author' since the protocol may not (and the full review will not) have been published at the time of registration. This requirement for a single contact person should encourage review teams to nominate one person to this role and so help avoid a review being registered more than once.

### Scope for inclusion

The long term aim is to have broad inclusion criteria for PROSPERO, such that any systematic review that has a health related outcome will be eligible. However, to reach this aim without making the process too complex or time consuming, a stepped approach is being taken. The initial focus for inclusion is on systematic reviews of the effects of interventions and strategies to prevent, diagnose, treat, and monitor health conditions, for which there is a health related outcome. This includes systematic reviews undertaken before and after clinical trials to help design the trial or to place the results in context [7]. The inclusion of other reviews will be phased in over time.

Systematic reviews that are regarded as 'rapid reviews' will be accepted if they meet the inclusion criteria and researchers can complete the application within the time frame of the review and in accordance with the requirements of PROSPERO.

Scoping reviews and reviews of reviews are not being included at this time, but this decision will be re-considered in the future. The decision to exclude these types of knowledge syntheses was based on practical considerations: it is not clear if the initial registration template will be suitable for much broader types of knowledge syntheses where the methods vary and may not be as well defined as those that use well-accepted systematic review methodology.

Reviews of methodological issues will not be included in PROSPERO as the findings are likely to relate to recommendations about changes in methods rather than direct effects on health outcomes [8]. Methods reviews often cross boundaries between health and other areas, and like other types of knowledge syntheses, are also likely to require a different data entry structure. A centralized database of such reviews would be useful, but is outside the current aims and remit of PROSPERO. Likewise, systematic reviews of animal studies are excluded as they involve studies with different methodologies and objectives.

The inclusion of protocols for Cochrane Reviews is desirable to ensure a comprehensive overview of ongoing systematic reviews. To minimize additional work for authors of Cochrane Reviews, an electronic mechanism for their automatic upload from *The Cochrane Library *is being developed. Contact authors will simply be asked to verify that the information has been transferred accurately to the PROSPERO database. To avoid future duplication, Cochrane Reviews are therefore not registered independently on PROSPERO.

### Timing of registration

As registration requires the completion of a minimum dataset, it can only take place after key issues have been considered, preferably as part of the development of the review protocol. For PROSPERO to achieve its aim of providing transparency and helping identify potential bias, registration should ideally take place before formal screening against inclusion criteria has begun, this being an early point at which bias could be introduced. However, the systematic review process is iterative by nature and some experimentation with searching is likely to be essential in developing the review. It also has to be recognized that researchers are often aware of some of the potentially eligible studies, and have an opinion on whether they are likely to include or exclude these, some time before they start formal screening.

Registering a review too soon might lead to multiple amendments to records as the protocol and the plans for the review are finalized; registration late in the process may mean that the aim of publishing methods before any results are known is not achieved. A practical approach to the timing of registration has been taken, initially. Registrants are asked to indicate the stage of progress of the review at the time of registration, and at any subsequent revisions, by selecting the relevant stage from a list, with the option of adding further information in a free text field. All records and revisions are automatically dated when published in the register.

In recognition that authors of reviews that are already underway during PROSPERO's first year might wish to register them, systematic reviews that have not progressed beyond the point of completing data extraction are being considered for inclusion. The issue of timing of registration will be reviewed as part of a planned evaluation of the register in 2012.

### Registering a review

**Figure 2:**
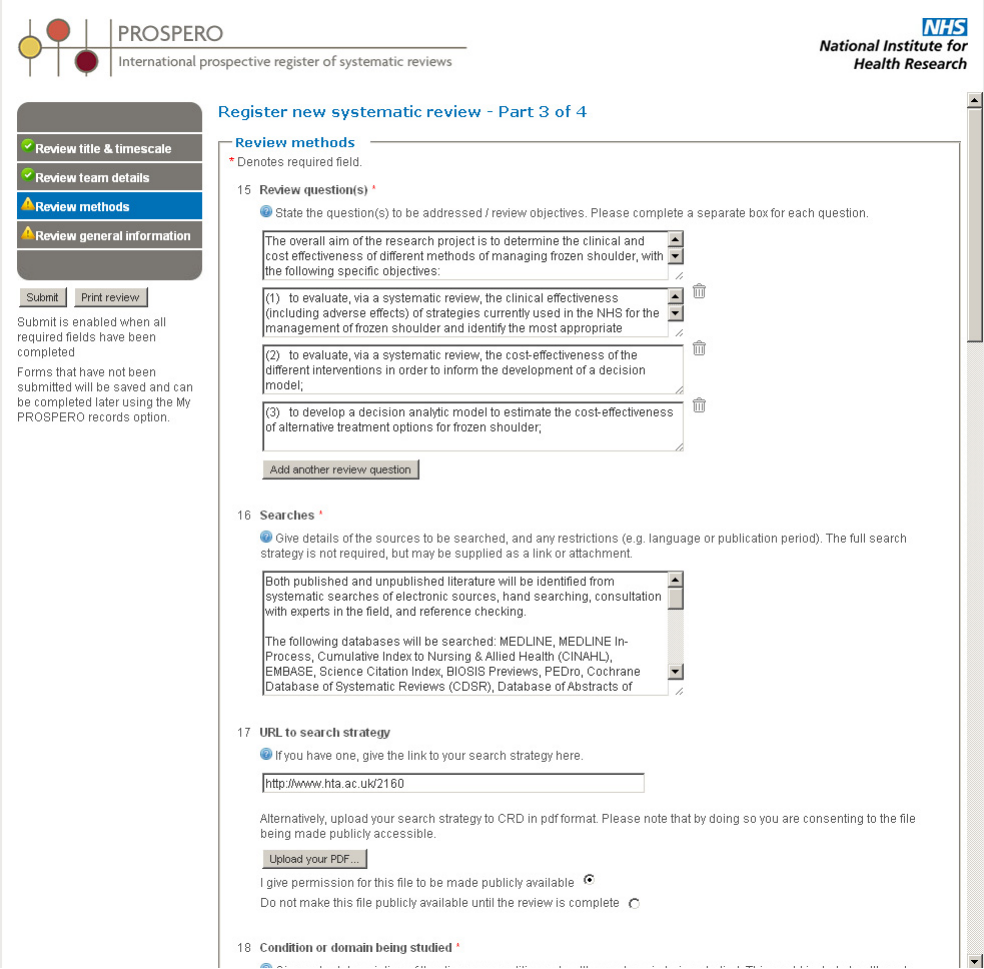
**PROSPERO registration form**.

Registrants need to 'Join' PROSPERO to obtain a username and password, which are then used to sign in and activate the 'Register a review' option. Selecting this option opens a page detailing a summary of the inclusion criteria, to help users to avoid wasting time on inappropriate submissions. Once registrants are satisfied that their review fulfills the inclusion criteria, a single click opens a new electronic registration form. (Figure 2)

There are four sections to the form: title and timescale; review team details; methods; and general information. All the 'Required' fields in each section are indicated by an asterisk (*) in the on-line form and below, and these must be completed before the registration can be submitted. A registration application can be saved and returned to at any time, to add or edit information before submission. Information can be entered by typing directly into the form or by pasting from another document. Once all the required information has been provided, the 'Submit' option is activated.

### The PROSPERO registration form

#### 1. Review title and timescale

The first section in a PROSPERO entry asks for the title of the review in English* and the original language if this is not English. Registrants are asked to give the anticipated or actual start date* and the anticipated completion date for the review*. Unless 'fixed' by a funder, these dates can be difficult to estimate. However, they are operationally necessary for scheduling automatic updates and reminders, as well as for the integrity of the record. The dates can be revised at any time by submission of an amendment. The Delphi consultation revealed some differences of opinion about when a review 'starts'. For PROSPERO purposes this is considered to be when screening studies for inclusion begins, although it is recognized that a large amount of essential work takes place before this.

#### 2. Review team details

This section includes address, phone, and email* contact details for the Named contact*. These fields are automatically completed from the 'Join' information, but can be edited. For example, information in optional fields can be deleted so that it does not appear in the public record.

The organizational affiliation of the review*, funding sources/sponsors* and conflicts of interest* were categorized as essential details by respondents to the consultation. The names of review team members and their organizational affiliations and information about collaborators were considered by respondents to the Delphi survey to be useful indicators of the range of skills and experience of those undertaking the review, but not essential to the register.

#### 3. Review methods

There are 15 fields to capture the review methods, 12 of which are 'Required'*:

##### Review methods fields

• Review question(s)*

• Searches*

• URL to search strategy

• Condition or domain studied*

• Participants/population*

• Intervention(s), exposure(s)*

• Comparator(s)/control*

• Types of study to be included initially*

• Context

• Primary outcome(s)*

• Secondary outcomes*

• Data extraction, (selection and coding)

• Risk of bias (quality) assessment*

• Strategy for data synthesis*

• Analysis of subgroups or subsets*

The structure aims to facilitate data entry for registrants while also providing users of the database with consistent, clear access to the planned methods for a review. Registrants are asked to provide sufficient detail to allow comparison of planned methods with the subsequent published review. The information to be provided will vary according to the type of review and the topic, and not all fields will be relevant to all reviews (with 'not relevant' being an acceptable response, where appropriate). Within the registration form, brief instructions are given for what is required for each field and users can access expanded guidance with examples either within the information tabs for each field or from the 'About' pages on the PROSPERO site.

The review methods fields were agreed through the Delphi consultation and are based on the protocol requirements for a variety of reviews of the effects of interventions, ranging from a straightforward comparison (for example, a drug versus a placebo) to the assessment of complex interventions (for example, smoking cessation), hence the inclusion of a field such as Context. To achieve the long term aim of a broad scope for PROSPERO, it is anticipated that other templates may need to be developed in consultation with experts in particular fields (such as for reviews of qualitative research).

#### 4. Review general information

Additional general information about the type of review, language, countries involved, other registration details, dissemination plans, keywords and existing reviews on the same topic by the same authors were identified as useful but not essential during the consultation.

Respondents to the consultation suggested that other registration details be recorded, but that these should not be mandatory. This would allow appropriate cross-linkage, and help avoid registration duplication. This information has been incorporated into the registration form as one of the 18 optional fields.

Respondents also agreed that where a protocol had been published for a review and was publicly accessible, the citation and URL should be included in the PROSPERO record. The challenges and opportunities for publishing protocols vary across different areas of health and social care, with limited scope up to now to publish review protocols outside *The Cochrane Library*. However, the launch of the journal *Systematic Reviews *should improve this situation.

While publication of review protocols is recommended and encouraged, submission of a review to PROSPERO is not dependent on it. 'Publication' is considered in a wider sense than inclusion in a peer reviewed journal. For example, protocols made available on organizational websites are acceptable and can be linked to from PROSPERO. Alternatively, registrants can submit a pdf of their protocol, which will be hosted on a CRD web server and linked to from within the register record. In either case, the named contact is responsible for the integrity and maintenance of the protocol. If a protocol is not available in a published record, users of PROSPERO are advised to get in touch with the named contact for any further information they wish to obtain about the review.

The Current review status* field is an administrative requirement to indicate the progress of the review through the process from design to full review.

Registrants can add any further information they think relevant to their registration in a free text field. The last field is for recording details of the final report or publication when the review has been completed.

### Administration of submissions

On submission, registrants receive an automated email confirming receipt and outlining the administration process. Submitted application forms are checked for eligibility for PROSPERO, which includes consideration of the current stage of review. Forms are also examined for clarity of content, for example whether: the information provided makes literal sense; the information has been entered in the correct field; the information given is not contradictory; or only partial information is provided in a required field. Submissions are approved and published on the register, returned to the applicant for clarification, or rejected. The checks made do not constitute peer review or imply approval of the methods proposed for the review being registered.

Applications are reviewed within five working days of submission and details of the final decision are sent to the named contact in a confirmation email. In the case of accepted records, a unique ID number is given in the email. All records published in PROSPERO remain permanently available through the register.

### Recording protocol amendments

Protocol development is an iterative process and legitimate changes and amendments to the registration record may be necessary. It is particularly important for transparency to document and justify major changes to methods, particularly those which could be seen as potentially introducing biases through increased knowledge of potentially eligible studies, resulting, for example, in the narrowing of objectives or the addition of new outcome measures.

Registrants can access and update their records at any time via a 'My PROSPERO records' page, except during the PROSPERO administration phase, when access to the record is locked. The named contact is tasked with recording any major changes or substantial amendments to the planned methods in the PROSPERO record. This is done by making the necessary changes in the record, updating the stage of review and re-submitting it. A 'Revision note' facility requires a brief outline of the changes and the reason for making them to be recorded. This is made available in the public record, as part of the audit trail for the PROSPERO entry.

The most recent version of a record appears in the public interface, with previous versions marked as 'Archived' and made accessible through dated links on the record page.

### On completion of a registered review

There was strong support in the Delphi consultation for PROSPERO to include publication details or details of where unpublished results could be viewed, once the review is completed. It was considered that such links would be hard to maintain, but the consensus was that this would be necessary to provide a complete thread for a systematic review. However, there was also concern that the register should not become a new database of completed reviews. The addition of details of the completed review is an option available to registrants. There are currently no plans for the PROSPERO administration team to be responsible for identifying publications or adding links within PROSPERO records.

Email reminders are sent to the named contact on the completion date entered in the PROSPERO record, asking for an anticipated publication date (or revision to the completion date). The named contact is prompted to add a statement if the review will not be published, including brief details of the reason. This can be entered in the final report/publication field.

If a registered review is not to be completed, the option of 'Abandoned' can be selected and brief details of the reason why recorded in a free text field, for display in the public record.

If a registered review is completed and a critical abstract for its publication is included in the Database of Abstracts of Reviews of Effects (DARE), a link to the DARE abstract will be added to the PROSPERO record.

As part of the consultation, participants were asked about the inclusion of summary results in the PROSPERO record, given that a sizeable proportion of initiated systematic reviews are never published [9]. Some major concerns were expressed. These included that publishing results on the register could jeopardize subsequent peer review publication; and that, as publication can take a long time, it may be seen as an alternative and delay or prevent more formal publication by some review teams (for example, where their funding has ended). It was also thought that if researchers had not published the review, it was likely they would have lost interest and would not provide this information anyway. Of more concern was the inability to check the validity of the data posted, and the potential lack of context for it, which might be misleading if users of PROSPERO read the record and not the full publication of the review. In light of these concerns, it was decided that summary results would not be included in PROSPERO records, at this time.

### Updating an existing review

The intention of including protocol details for updates to existing reviews prompted a discussion on how to deal with these updates, and how to decide if the modifications to an existing protocol constitute a new review rather than an update. The advisory group agreed on the following definitions, which are included in PROSPERO's guidance notes:

#### What is an update of a review?

Updating a systematic review is a discrete event during which efforts are made to identify and incorporate new evidence into a previously completed systematic review [10].

An 'update' may be any modified version of a review that includes the findings of a more recent search than the previously completed version of the review. It can still be considered an update even if the new search reveals no additional studies. Any newly identified studies should be assessed and, if appropriate, incorporated into the updated review. An update might also be an opportunity to conduct new analyses or add additional information to the review.

#### What constitutes a new review rather than an update?

It can be difficult to decide whether an update to a review is in fact a new review. There is little published guidance on this. PROSPERO adopts a pragmatic approach. If changes to the review questions or methods are so substantial that they require major changes to the original protocol, this should be regarded as a new review rather than an update.

Examples that would constitute a new review:

• addition of new treatment comparisons, for example, direct comparison of different drugs, when the old review included only comparisons of drug with placebo

• substantial changes to the population being studied, for example, adding adults to a review that was previously restricted to children

• exclusion criteria in the old review become inclusion criteria in the new review

• introduction of new analysis techniques, for example, a switch from aggregate data meta-analyses to individual participant meta-analyses.

Updates of registered reviews will retain the original number and the version history will be available, which will mean that links to the full audit trail and the existing review will be readily accessible to users.

## Conclusions

### Current and future developments

PROSPERO was launched in February 2011 by the UK Health Minister Lord Howe and at an international meeting in Vancouver, Canada organized by the Canadian Institute for Health Research (CIHR).

Initial publicity efforts have gone into raising awareness of PROSPERO among those commissioning and undertaking reviews. There has been an enthusiastic international response to the development of PROSPERO, alongside support for the principle of systematic review protocol registration from organizations, including the International Network of Agencies for Health Technology Assessment (INAHTA), The Cochrane and Campbell Collaborations and the Guidelines International Network (G-I-N). A number of commissioning organizations, such as the UK National Institute for Health Research (NIHR) and the Canadian Institute of Health Research (CIHR), are making registration a requirement for all their grant holders who are undertaking relevant systematic reviews.

Public Library of Science journals and the *Systematic Reviews *journal support the prospective registration of systematic reviews and their instructions to authors ask that the registry number be included in the abstract of the reports of all prospectively registered systematic reviews. Other journals are being encouraged to follow suit.

**Figure 3:**
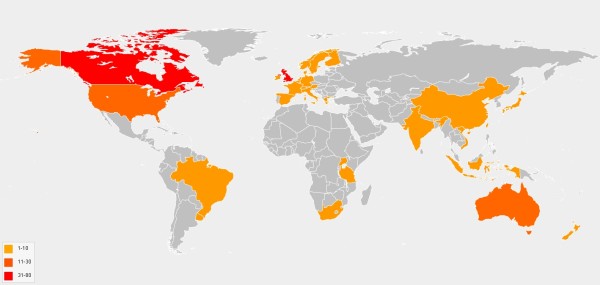
**Countries where registered reviews are being undertaken**.

In October 2011, eight months after launch, PROSPERO contained 200 records of systematic reviews being undertaken in 26 different countries (Figure 3) on a diverse range of interventions.

Feedback from users is welcome (to crd-register@york.ac.uk) as part of an ongoing process of improvement and refinement. A detailed evaluation of the registration process is planned for early 2012. The findings of this will be used to make an initial assessment of PROSPERO's fitness for purpose and guide the next stages in its ongoing development.

## Competing interests

The authors declare that they have no competing interests.

The development of PROSPERO is being funded by the NIHR Centre for Reviews and Dissemination at the University of York, England.

## Authors' contributions

All the authors made substantial contributions to the Delphi consultation exercise and the subsequent development of PROSPERO. AB managed the acquisition of the data; all the authors contributed to the analysis and interpretation of the data and conversion into the working register, PROSPERO. AB produced the first draft of the article and MC, GD, DG, DM, MP, and LS critically commented. All the authors have read and approved the final version being submitted.

## Authors' information

All the authors are members of the PROSPERO advisory group and have been since the inception of the register.
